# Structure, Chemical Analysis, Biosynthesis, Metabolism, Molecular Engineering, and Biological Functions of Phytoalexins

**DOI:** 10.3390/molecules23010061

**Published:** 2017-12-28

**Authors:** Philippe Jeandet

**Affiliations:** Research Unit “Induced Resistance and Plant Bioprotection” EA 4707, SFR Condorcet FR CNRS 3417, Faculty of Sciences, University of Reims Champagne-Ardenne, PO Box 1039, 51687 Reims CEDEX 2, France; philippe.jeandet@univ-reims.fr

Plants in their natural environment are facing large numbers of pathogenic microorganisms, mainly fungi and bacteria. To cope with these stresses, plants have evolved a variety of resistance mechanisms that can constitutively be expressed or induced. Phytoalexins, which are low-molecular-weight antimicrobial compounds produced by plants as a response to biotic and abiotic stresses, take part in this intricate defence system. This special issue is the continuation of that published in 2015 entitled “Phytoalexins: Current Progress and Future Prospects” through http://www.mdpi.com/journal/molecules/special_issues/phytoalexins-progress.

Phytoalexins display a wide range of properties as antifungal compounds in various plants or preventing actions against human diseases as antioxidant, anticancer and cardioprotective agents as well as being supposed to act positively in neurodegenerative diseases such as Alzheimer’s and Parkinson diseases. These compounds have been the subject of numerous studies over the last two decades. Thirteen research and review articles have been published in this special issue: four articles concern the biosynthesis of phytoalexins as a response to biotic and/or abiotic elicitors capable of inducing their production in plants [1,2,3,4], two articles describe methods for phytoalexin analysis in complex matrices [5,6], one article reports on phytoalexin metabolism by fungi [7], and six articles focus on the biological activity of phytoalexins [8,9,10,11,12,13].

By definition, phytoalexins are non-constitutive compounds produced by plants solely as a response to potentially pathogenic microorganisms or a large number of biotic and chemical elicitors. In the work of Woźniak et al. [1], the ability of a chemical elicitor, lead employed at various doses, or a biotic factor, pea aphid infestation, to act on the signaling pathways (salicylic acid, SA and abscisic acid, ABA production) of pea seedlings was studied. Regulation of the level of these two signaling molecules was also assessed upon cross interactions between the abiotic factor (lead) and the biotic factor (aphid infestation). The elicitor-mediated increases of the SA and ABA pathways in pea resulted in a strong induction of the biosynthesis of the phytoalexin pisatin. In the article of Farrell et al. [2], two distinct elicitors were also used to enhance the production of glyceollin I, a phytoalexin from soybean. Combination of the chemical elicitor, silver nitrate (AgNO_3_) with the wall glucan elicitor (WGE) from the pathogen *Phytophthora sojae* was shown to have an additive effect on the induction of glyceollin production in soybean, reaching up to 745 µg/g tissue. Both elicitors act by distinct mechanisms. WGE upregulates the genes working on the isoflavonoid and glyceollin pathways while AgNO_3_ increases hydrolysis of 6″-*O*-malonyldaidzein to form daidzein, an intermediate in the glyceollin pathway.

Oligogalacturonides (OGs) are well known potent stimulators of the plant immune system. In the work of Selim et al. [3], the eliciting activity of two OG fractions of varying polymerization degrees, one non-acetylated and one 30% acetylated, was determined in pea against *Aphanomyces* root rot. Significant root infection reductions were observed in both cases. The OG-mediated increased resistance of pea to *Aphanomyces* root rot was linked namely to an upregulation of the genes involved in the phytoalexin pisatin pathway (phenylalanine ammonia lyase, chalcone synthase, and isoflavone reductase).

Study of the biological properties of phytoalexins is hampered by their limited supply and the impossibility to recover them in sufficient amounts by conventional plant extraction procedures or chemical synthesis. The use of biotechnological systems could thus represent powerful methods for the production at large-scale of these compounds. In the article of Tisserant et al. [4], hairy root cultures of grapevine obtained after transformation with *Rhizobacterium rhizogenes* were used for obtaining high-purity stilbene phytoalexins. A significant accumulation of resveratrol, piceid, and ε- and δ-viniferins was observed both in the fresh tissues and the extracellular medium as a response to a combination of two elicitors, methyljasmonate and cyclodextrins.

As phytoalexins are naturally occurring compounds of a very diverse nature, highly specific qualitative and quantitative analytical techniques are thus needed for their accurate determination in complex matrices such as biological fluids, plant extracts, or plant cell cultures. In the work of Hurtado-Gaitán et al. [5], a method coupling ultra-high-performance liquid chromatography to triple-quadrupole mass spectrometry operated in the multiple reaction monitoring mode was developed for the detection and the quantitation of five stilbene phytoalexins from grapevine (trans-resveratrol, trans-piceid, trans-piceatannol, trans-pterostilbene, and trans-ε-viniferin). The applicability of the technique was verified in various matrices including cell culture extracts and red wine, and the method was also used to follow the enzymatic conversion of trans-resveratrol to trans-piceatannol in the presence of NADPH substrates and grape protein extracts. In the work of Hwan Hwang et al. [6], four phytoalexins found in Compositae species, three isoflavonoid-type phytoalexins (linarin, luteolin, and apigenin) and one phenylpropanoid-related phytoalexin (chlorogenic acid) were analyzed and quantized by ultra-performance liquid chromatography. The technique employed led to a good resolution of the four phytoalexins with a reasonable analysis run time (14 min). Upstream applications of this method resulted in the determination of the antioxidant activity of the four phytoalexins.

The ability of a pathogenic microorganism to detoxify the phytoalexins to which it is exposed is an essential component of the cross talk between plants and pathogens. In the article of Pedras et al. [7], research on phytoalexin detoxification inhibitors, the so-called PALDOXINS, was developed. Work has focused on inhibitors of brassinin oxidase which is an inducible fungal enzyme from the plant pathogen, *Leptosphaeria maculans*, catalyzing the detoxification of the phytoalexin brassinin to indole-3-carboxaldehyde and *S*-methyl dithiocarbamate. It is suggested that quinoline-derived compounds, especially 3-ethyl-6-phenylquinoline, display the highest inhibiting activity of the brassinin oxidase.

Beside their antifungal properties in plants, phytoalexins show preventing activities against human diseases as antioxidant, anticancer, cardioprotective, antibacterial, and antifungal agents. Six articles report here on the biological implication of phytoalexins in human diseases [8,9,10,11,12,13].

In the work of Moon et al. [8], the antifungal activity of natural and synthetic coumarins, which are phenylpropanoid-derivated phytoalexins, was evaluated against *Aspergillus flavus*. This mold is responsible for the production of various aflatoxins, considered to be the most important carcinogenetic agents of natural origin. Among 26 tested coumarins, five compounds displayed potent antifungal and antiaflatoxigenic activities against *A. flavus*. The 4-hydroxy-7-methyl-3-phenyl coumarin especially showed a 50% inhibition of the fungal growth at a concentration of 100 µg/mL. Most interestingly, coumarins displayed remarkable inhibition effects at 10 µg/mL on the production of aflatoxins B1 and B2, being the activity of the 4-hydroxy-7-methyl-3-phenyl coumarin correlated with the downregulation of several genes (*aflD*, *aflQ*, *aflR*, and *aflK*) working on the biosynthetic route to aflatoxin.

Phytoalexins exert some inhibiting activity against a range of bacteria or fungi implicated in human diseases such as skin infection, candidiasis, gonorrhea, and respiratory tract infections. In the work of Lee et al. [9], the antibacterial activity of pterostilbene, a dimethylated phytoalexin derived from resveratrol, in combination with the antibiotic gentamicin was evaluated against six strains of Gram-positive and -negative bacteria. Results evidenced a synergistic action between the phytoalexin and the antibiotic against *Staphylococcus aureus* ATCC 25923, *Escherichia coli* O157, and *Pseudomonas aeruginosa* 15,442. Growth of the tested bacteria was completely inhibited by the synergistic action of pterostilbene and gentamicin within 2–8 h treatment with half of their minimum inhibitory concentrations.

Numerous phytoalexins have been reported to exhibit significant anticancer, chemopreventive, and antiproliferative activities. In the article of Chripkova et al. [10], the antiproliferative effects of indole phytoalexins including brassinin, homobrassinin, camalexin, and their synthetic derivatives have been reviewed. Mechanisms of their anticancer actions include induction of apoptosis and cell cycle arrest, inhibition of neovascularization, and modulation of the signaling pathways associated with malignant transformation or cell survival. Search for synthetic derivatives of indole phytoalexins such as the 2-amino derivatives of spiroindoline phytoalexins displaying high anticancer features was also described. In the work of Nivelle et al. [11], a new dimer of resveratrol called labruscol has been purified and identified from grapevine cell suspensions of *Vitis labrusca* L. cultivated in a 14 L bioreactor. The antiproliferative activity of labruscol was demonstrated, this compound exerting almost 100% of cell viability inhibition of the human skin melanoma cancer cell line HT-144 at a dose of 100 µM within 72 h of treatment. Moreover, at the very low concentration of 1.2 µM, labruscol showed a 40% inhibition of cancer cell invasion, an activity not displayed by resveratrol. It thus seems that labruscol possesses complementary properties of resveratrol in particular regarding cell invasion, suggesting its utilization in combination with resveratrol to improve its antiproliferative capacities. In the work of Aires et al. [12], the identification of microRNAs was described following treatment of human primary fibroblasts with resveratrol. This study focuses on the relation between resveratrol treatment and deficiency in Carnitine-Palmitoyl Transferase-2 (CTP2), a mitochondrial enzyme involved in long-chain fatty acids entry into the mitochondria for their β-oxidation and energy production. It has indeed already been shown that resveratrol treatment restores normal fatty acid oxidation rates in patients harboring CPT2-gene mutation. Data resulted in the identification of several microRNAs displaying altered expression levels in fibroblasts either in the presence or absence of CPT2 or in the presence or absence of resveratrol stimulation. In addition, putative target transcripts of the microRNAs were described, suggesting that their gene products are important for the detrimental effects of CPT2 and the beneficial effects of resveratrol.

Although resveratrol has been shown to prevent the proliferation of malignant cells, the molecular mechanisms mediating resveratrol specific effects on lymphoma cells remain unknown. To answer this question, Jara et al. investigated cell survival and gene expression in the Burkitt’s lymphoma cell line Ramos upon treatment with resveratrol [13]. The data obtained suggest that resveratrol displays significant anti-proliferative and pro-apoptotic activities on those cells, modulating the expression of several genes implied in the apoptotic process as well as inducing the DNA damage response and DNA repairing. From a mechanistic point of view, the data clearly correlated the decrease in malignant cell survival with the activation of apoptotic markers such as caspase 3 and fragmented poly(ADP-ribose) polymerase 1 in a dose-dependent manner. Moreover, expression of the pro-apoptotic genes *Noxa* and *Puma* was increased in a time-dependent fashion after 1 h and 3 h of resveratrol treatment, but no effect was observed on the expression of the *Fas* gene. Additionally, resveratrol induced significant increases in proteins necessary for the initiation of the DNA repair pathway.

All these articles thus highlight the central role played by phytoalexins in plant–microbe interactions as well as in human diseases. This special issue is accessible through the following link: http://www.mdpi.com/journal/molecules/special_issues/phytoalexins.
